# Evaluation of the Mechanical Properties and Drilling of Glass Bead/Fiber-Reinforced Polyamide 66 (PA66)-Based Hybrid Polymer Composites

**DOI:** 10.3390/ma15082765

**Published:** 2022-04-09

**Authors:** Recep Demirsöz, Nafiz Yaşar, Mehmet Erdi Korkmaz, Mustafa Günay, Khaled Giasin, Danil Yurievich Pimenov, Muhammad Aamir, Huseyin Unal

**Affiliations:** 1 Department of Mechanical Engineering, Karabük University, Karabük 78050, Turkey; recepdemirsoz@karabuk.edu.tr (R.D.); mgunay@karabuk.edu.tr (M.G.); 2 Yenice Vocational High School, Karabük University, Karabük 78050, Turkey; nafizyasar@karabuk.edu.tr; 3 School of Mechanical and Design Engineering, University of Portsmouth, Portsmouth PO1 3DJ, UK; khaled.giasin@port.ac.uk; 4 Department of Automated Mechanical Engineering, South Ural State University, Lenin Prosp. 76, 454080 Chelyabinsk, Russia; danil_u@rambler.ru; 5 School of Engineering, Edith Cowan University, Joondalup 6027, Australia; m.aamir@ecu.edu.au; 6 Department of Metallurgical and Material Engineering, Faculty of Technology, Sakarya University of Applied Sciences, Kemalpaşa 54187, Turkey; unal@subu.edu.tr

**Keywords:** polymer composites, drilling, glass bead, glass fiber, thrust force, surface texture

## Abstract

In this study, mechanical testing of glass bead (GB), glass fiber (GF), and hybrid (GB/GF) composites was carried out. Following that, drilling tests were undertaken on glass bead/fiber-reinforced hybrid Polyamide 66 (PA66) polymer composites. The purpose of this study is to determine the mechanical properties of the cutting elements and the effect of cutting parameters (spindle speed and feed rate) and reinforcement ratios on thrust force and surface roughness (Ra). The contribution of the cutting parameters to the investigated outcomes was determined using statistical analysis. Optical microscopy and scanning electron microscopy (SEM) was used to inspect the hole quality and damage mechanisms. The results revealed that the feed rate was the most contributing factor to thrust force (96.94%) and surface roughness (63.59%). Furthermore, in comparison to other hybrid composites, the lowest R_a_ value was obtained as 0.95 µm in samples containing 30% GB, while the R_a_ value was 1.04 µm in samples containing 10% GF + 20% GB. Polymer PA reinforced with 30% GF had the highest strength, modulus of elasticity, impact strength, and hardness.

## 1. Introduction

Polyamide, epoxy, polyester, phenolic, and vinyl ester are used as a matrix in polymer composite materials due to their high strength, low density, excellent chemical stability, and superior corrosion strength [1,2]. However, these materials are reinforced with different types of fibers [3] such as glass, carbon, basalt, and aramid, as they show comparatively low hardness and propensity to creep at high temperatures [4,5]. Glass beads (solid glass micro-beads or glass beads) are suitable for aviation and marine applications thanks to their excellent mechanical and thermal properties [6,7]. In addition, they have a strong filling ability, low and good dispersed internal tension in products, and are easily processed for filling materials. Thanks to the smooth spherical surfaces of these microparticles, there is no stress concentration at the interface between the reinforcements and the matrix. They are favored as reinforcements, and particularly when combined, properties such as isotropy or low melt viscosity are critical. Composites that contain glass beads are commonly used in the building, spaceflight, and aviation industries [8]. Various glass bead-reinforced polymer-based composites are used to manufacture functional parts with special mechanical properties using additive manufacturing technologies [9]. Alternatively, the use of fiber-reinforced composite materials is developing rapidly [10], especially in the aerospace, aviation, automotive, sports products, and marine industries [11,12]. Composite components produced by molding are machined using the milling and drilling process for assembly and to bring the part to the desired dimensional tolerances. [13]. The machinability of fiber-reinforced composites differs from fiber-only materials [14,15]. The mechanical–thermal characteristics of these materials have a significant effect on machinability [16]. In addition, the machinability of fiber-reinforced composites depends on the characteristics of the cutting tool (material and geometry) and machining parameters [17,18]. Since fiber-reinforced composite materials are anisotropic, their formability is different from that of homogeneous materials such as metals [19]. The anisotropic nature of composite materials affects their machinability. Other factors, such as the type of reinforcement and fiber volume fraction, play a significant role in the quality of the machined part. Tool wear can be a problem when machining composites, especially when abrasive fibers are used in the composite. Therefore, excellent wear-resistant cutting tools are recommended when machining composite materials [20]. The hole-making process, which is accomplished using drilling, accounts for 40% of all machining processes [21]. The drilling of composite materials is common, but certain drilling-induced damage phenomena such as delamination, heat-affected zones, and fiber rupture might occur. These unfavorable damage phenomena can cause serious problems in the machined composite materials, and therefore should be eliminated or minimized. To prevent this, a proper selection of cutting parameters and cutting tools should be considered. Other parameters are important, but those two have a significant effect on the quality and precision of the machined parts [22,23].

A good number of studies in the open literature investigated the drilling of glass fiber-reinforced polymer (GFRP) composites in the past. Latha et al. used the Taguchi method and multiple regression analysis to model delamination during the drilling of GFRP composites using carbide drills. Their results showed that the feed rate and drill bit diameter were the most prominent input factors that can influence delamination [24]. Krishnaraj et al. performed a survey on the optimization of machining parameters when drilling thin carbon fiber-reinforced polymer (CFRP) laminates. It was emphasized that the circularity of the hole is the main factor, while the feed rate was the most influential factor on the thrust force, delamination, and hole diameter [25]. Gaitonde et al. examined the impacts of drilling parameters on thrust forces, hole diameters, and circularity on PA66 matrix–30% glass fiber-reinforced composites [26]. They found that the point angle of the drill can affect the thrust force and roundness of the hole. They recommended a point angle of 115° to minimize the thrust force when the drill had an 85°-point angle for minimal roundness error. Fıçıcı and Ayparçası found that the cutting tool material can affect the surface roughness when drilling 30% GFR polypthalamide matrix composite. They found that carbide drills produced holes with lower surface roughness than those produced using high-speed steel (HSS) drills [27]. Palanikumar et al. examined the cutting parameters that affect the thrust force when machining GFR polypropylene composites [28]. They found that the rise in the feed speed and drill diameter increased the thrust force. Ramesh et al. [29] found that the spindle speed is an important parameter that influences the thrust force, while the feed rate and drill geometry/material affect delamination when machining pultruded and liquid composite. Their results showed that diamond-coated HSS drills reduced delamination among other types of cutting tools used. In addition, it was found that the hole surface roughness was influenced by the fiber orientation. Mudhukrishnan et al. [30] found that HSS tools experience rapid tool wear in comparison to carbide, which in return increases the thrust force and delamination. They stated that holes drilled in SiC had minimal delamination and the best hole surface quality. According to Mohan et al. [31], the most critical factors in drilling glass fiber-reinforced polymer (GFRP) composites are material thickness and cutting speed for hole entry delamination and material thickness, and feed rate for hole exit delamination. According to a study by Latha and Senthilkumar [32], low feed rates resulted in decreased thrust force, which resulted in delamination. Karnik et al. [33] discovered a direct relationship between the delamination factor and the feed rate and cutting speed. Similarly, it has been demonstrated that drills with a high point of angle promote delamination. On the other hand, the multiple fiber directions used in drilling in anisotropic CFRP composite laminates are reported to have varying degrees of machinability. Additionally, it has been shown that surface defects such as resin loss, fiber pullout, and matrix degeneration seen in holes contribute to the formation of uneven surface textures [34]. SiAlON drills manufactured using powder metallurgy were found to operate effectively when used to drill CFRP composites with different geometries by Çelik et al. [35]. While drilling with a small double-point angle reduces thrust force and delamination, the researchers found that the geometry degraded hole quality due to uncut fibers on the surface of holes.

Although numerous studies have been conducted on the mechanical properties of glass bead (GB)- and glass fiber (GF)-reinforced polymers, there are no published studies on the drilling of the hybrid glass bead and glass fiber composites. The purpose of this study is to close this gap by evaluating the effect of fiber ratio, tool material and shape, and cutting parameters (spindle speed and feed rate) on hole surface quality, thrust force, and tool wear. Additionally, the mechanical properties of the machined composites were compared and evaluated (tensile strength, impact strength, and hardness). 

## 2. Materials and Methods

**Figure 1:**
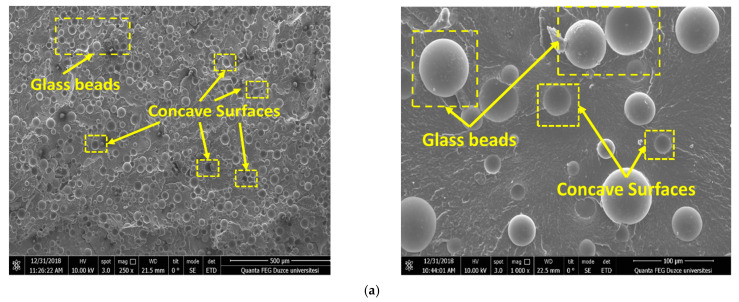

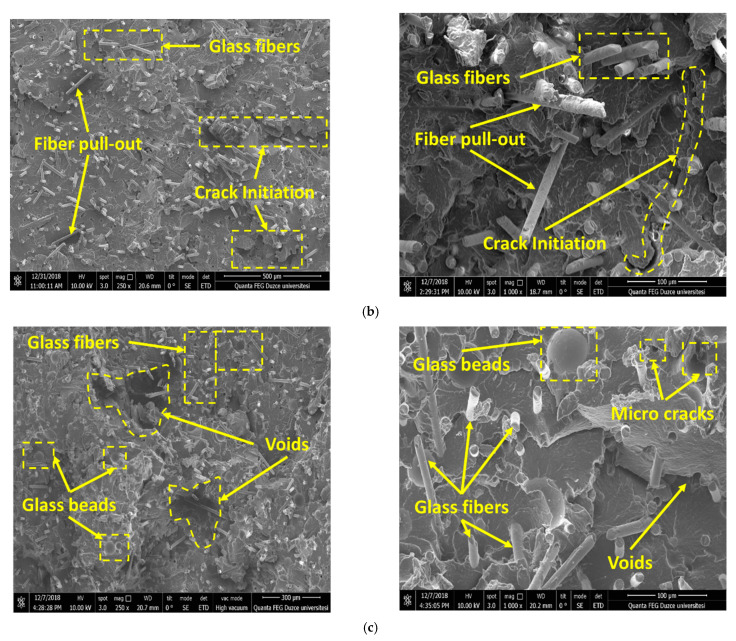
SEM views (250× to 1000× magnification) of composite materials with fractured surfaces (**a**) C1, (**b**) C2, (**c**) C3. (**a**) PA66 + 30% GB composite; (**b**) PA66 + 30% GF composite; (**c**) PA66 + 20% GF + 10% GB hybrid composite.

GFRP and GBRP composites with different reinforcement ratios were produced and their mechanical properties were investigated. The matrix material is Polyamide 66 (PA66), an engineering thermoplastic with excellent corrosion and abrasion resistance, self-lubricating properties, low impact resistance, and extremely high strength. The images provided below are of the supplied glass fiber (glass fiber_GF) and glass bead (GB) reinforcement elements within the composite. The PA2 short glass fibers (Sisecam Glass Fiber INC, Gebze, Turkey), with an average chopped length of 11 mm and a diameter of 4.5 mm, were reinforced with a coating of aminosilane, and the glass beads of 20 μm diameter were also coated with aminosilane (Microp is 1050-20-215, Sovitec, Fleurus, Belgium). The images of the supplied glass fiber (glass fiber_GF) and glass bead (GB) reinforcement elements within the composite are shown in Figure 1a–c, respectively.

**Figure 2:**
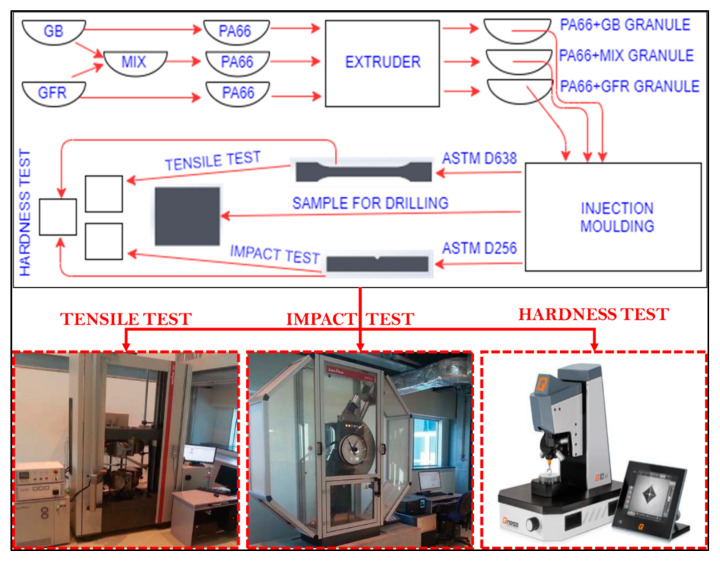
Manufacturing of polymer composites and mechanical test setup.

PA66 polymer-based composite materials containing different proportions of glass fiber and glass beads were first produced in granule form with a dual screw extruder. The extruder heater temperatures were adjusted between 260 and 295 °C from the feeding unit to the mold. After the granule production, the samples were printed in the injection machine (Ma2000 II/700e in Haitian INC, Barcelona, Spain) at an injection pressure of 130 bar and an injection speed of 66 mm/min. The mold temperature was maintained at 60 °C using a conditioner. The mechanical properties, for instance the tensile strength, impact strength, elasticity modulus, and hardness of the polymer composites, were measured as per ASTM. Tensile tests were conducted with a Zwick Z020 brand tensile machine at 50% humidity conditions and a tensile speed of 5 mm/min (Figure 2). The tensile specimens were injection molded in accordance with the ASTM D638 standard. Tensile and Izod impact samples using ISO 527 and ISO 180/1A standards, respectively, were prepared using an injection molding machine.

**Figure 3:**
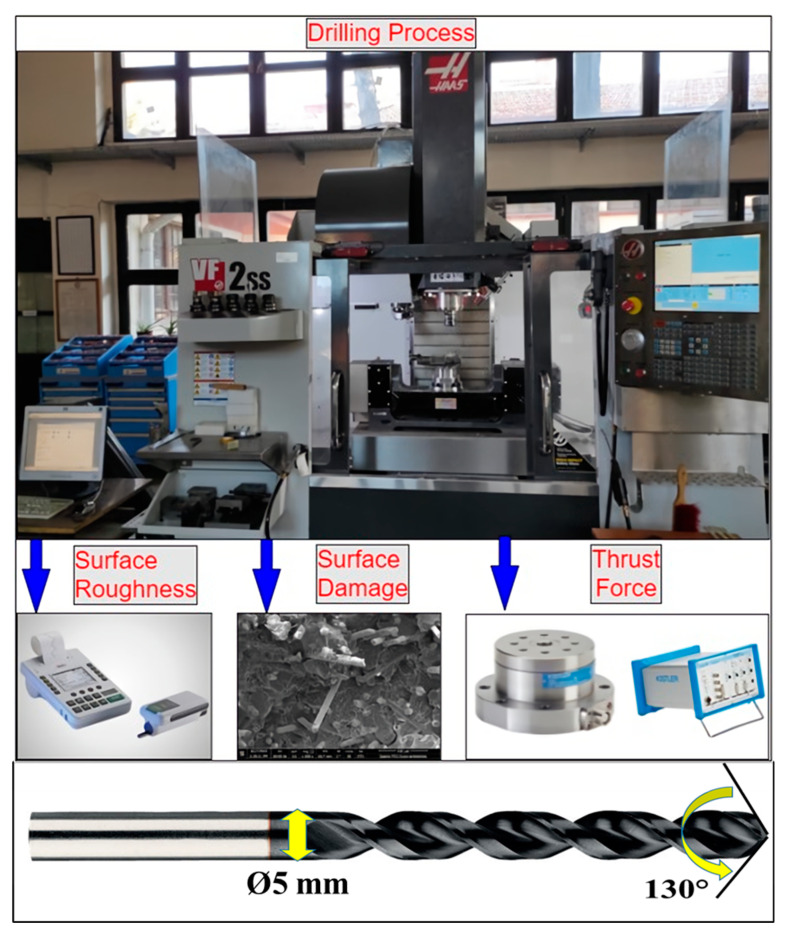
The machining system and flowchart.

In the second stage of the research, the machinability characteristics (thrust force and surface quality) of the polymer-based composites were evaluated via drilling tests. Drilling experiments were carried out using uncoated high-speed steel (HSS) drills supplied from KARCAN INC, Eskisehir, Turkey. The drills have a diameter of 5 mm, a tip angle of 120°, and a helix angle of 30° for the cutting edge (Figure 3). The experiments were performed at three different levels of feed rate and cutting speed determined by considering the cutting tool recommendation and relevant studies [36,37], as shown in Table 1. Drilling tests were performed under dry conditions without the use of coolant; nine experiments (three cutting speeds and three feed rates) for each composite material group (C1–C5) were conducted. The tests were conducted in a CNC setup located in the Manufacturing Engineering Department of Gazi University. An experimental system for the measurements and a flowchart are given in Figure 3.

The thrust force was measured using a Kistler 9257 piezo-electric dynamometer, a Kistler 5070-A type multichannel charge amplifier, and Dynoware 2825A-02-01 software from KISTLER INC, Eskisehir, Turkey. The surface roughness of the holes was measured to determine the impact of the type of cutting tool and cutting parameters. The Marsurf M 300 type profilometer from MAHR INC (Providence, RI, USA) was used to measure the hole surface roughness. A Gaussian filter was selected with a 0.25 mm cut-off and 1.25 mm sampling length. Surface roughness was measured four times by rotating the workpiece, and the average of the four readings is reported hereafter. SEM images of the internal hole walls were taken to inspect the damage mechanisms and surface integrity under different cutting parameters. Using the Minitab17 program, an ANOVA (analysis of variance) statistical analysis was conducted to evaluate the percentage contribution of the drilling parameters to the studied outputs. The ANOVA determines the statistical significance of the differences between groups of data. It works by assessing the degree of variance within groups using representative samples from each [38,39].

## 3. Results and Discussion

### 3.1. Mechanical Properties

The PA66 (C) polymer-based single reinforced composite samples are PA66 + 30% GB (C1) and PA66 + 30% GF (C2), and the hybrid composites are PA66 + 20% GF + 10% GB (C3), PA66 + 15% GF + 15% GB (C4) and PA66 + 10% GF + 20% GB (C5). The tensile strength, modulus of elasticity, impact strength, and hardness values of these materials are shown in Table 2.

**Figure 4:**
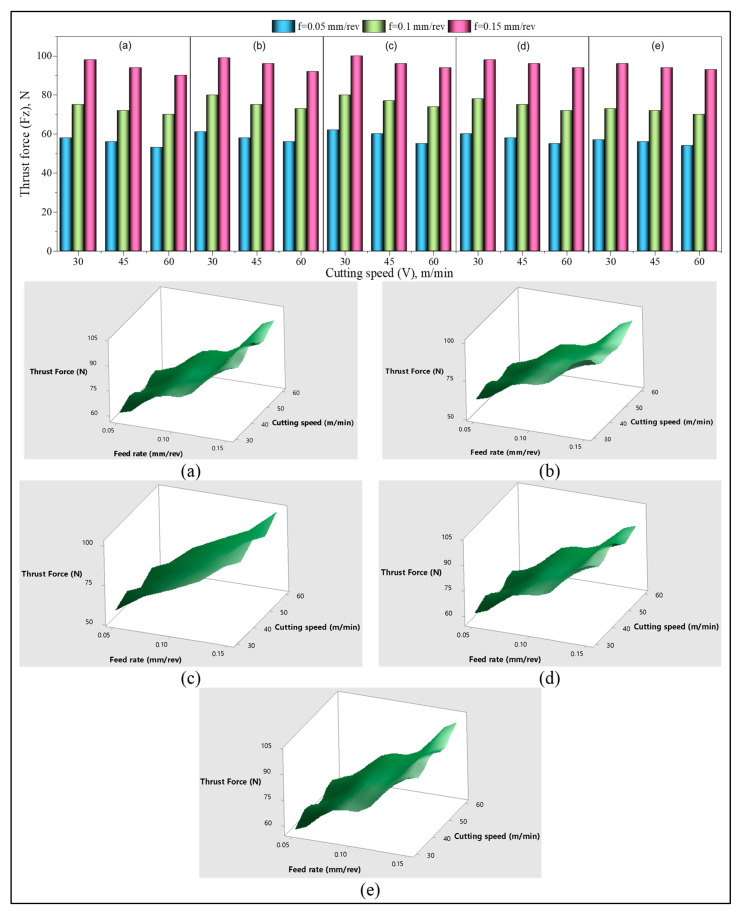
Thrust force (Fz) variation versus drilling parameters with 2D bar and 3D surface plots: (**a**) C1, (**b**) C2, (**c**) C3, (**d**) C4, (**e**) C5.

Since the loads to which composites are subjected are distributed through the interface to the matrix and fibers, a good interface bond is vital for improved mechanical properties. This fact can be confirmed by the further fracture rather than withdrawal of the fibers, as detected in the fractured surface SEM view taken from the rupture surface in the tensile test of the glass fiber-reinforced sample (C2) (Figure 1b). When Figure 1 is examined, it is observed that the glass fiber and glass beads within the PA66 polymer are (in general) homogeneously distributed. As can be observed from Figure 4a,b, the tensile strength of the C1 composite produced by adding 30% GB to the matrix decreased, while the elastic modulus increased. The reason for the 19% decrease compared to the tensile strength (73.6 MPa) of the PA66 polymer material is the interfacial micro-breaking that occurs around the glass bead particles during the loading of the composite [40]. The fact that the impact strength value of this composite (4.69 KJ/m^2^) is lower than the matrix material (5.84 KJ/m^2^) supports this inference, but it shows that the interface cohesion force between the matrix and the additive element is not satisfactory. C2 composite produced by adding 30% GF to the matrix has the best mechanical properties in terms of tensile strength, elastic modulus, impact strength, and hardness (Table 2). The tensile strength compared to PA66 increased by approximately 92% (142 MPa), and its elastic modulus increased by 186% (8.39 GPa). In addition, the C2 coded material has an impact strength of 7.46 and a hardness value of 82.9, indicating a strong interfacial cohesion between the PA66 and the GF reinforcement element, similarly to Ref. [41]. In other words, although GF-reinforced composites exhibit more isotropic behavior compared to GB-reinforced composites, a good interfacial bond creates a favorable effect for the impact strength by distributing the strain energy uniformly on the material. 

As can be seen from Table 2, when GB and GF were reinforced to PA66 polymer in different proportions, no considerable change occurred in the hardness of the composite materials. The highest hardness value was obtained in the C2 coded sample. The hardness decreased from C3 to C5 in the hybrid composites, and it can be said that this is due to the presence of GB reinforcement. However, it is seen that the tensile strength and elasticity modulus increase with certain proportions of GB and GF reinforcement in the hybrid composites (Table 2). Among these composites, the highest tensile strength and elasticity modulus were achieved in the sample PA66 + 20% GF + 10% GB (C3) with 127 MPa and 7.47 GPa, respectively. When the impact strength is examined, it is seen that there is a regular increase trend (Table 2). It was concluded that with 15% of the GB reinforcement ratio and 15% of the GF ratio, while the impact strength increased at a certain rate, the tensile strength decreased. This shows that GF and GB reinforcement members share the strain energy in certain proportions. It is attributed to the fact that GB does not create a stress concentration at the interface and increases the surface area to meet the load by filling the cracks formed in the matrix [42]. The highest impact strength was found to be 6.17 kJ/m^2^ in the PA66 + 10% GF + 20% GB (C5) sample. However, it was concluded that the fracture surfaces of PA66 matrix glass fiber-, glass bead-, and hybrid-reinforced composites are almost flat, and the increase in the reinforcing element ratio makes the composite material brittle. In the light of all of the outcomes, it is obvious that the mechanical properties of the PA66 polymer are improved by the addition of GB, GF, and GB/GF reinforcements, except for the tensile and impact strength of the GB-reinforced composite. 

### 3.2. Assessment of Thrust Force (F_z_)

Hole surface damages caused by the drilling of polymer-based composites are generally due to the thrust force that varies depending on the machining conditions. In addition, the damages caused during drilling can vary for the reinforcement element form and size. In this context, a comprehensive analysis of the thrust force generated by the drilling of GF- and GB-reinforced PA66 polymer is essential. Figure 4 indicates the thrust forces achieved when drilling glass bead (GB) with PA66 matrix, glass fiber (GF), and GB + GF hybrid-reinforced materials using HSS carbide drills with different cutting speeds and feed rates. When the graphs are evaluated, the F_z_ values tend to increase in general with 100% and 200% increases in the feed rate. Increasing the feed rate increases the uncut chip thickness [43]. This results in an increased power required for chip removal and, therefore, the F_z_ rises. [14,44,45]. There is a slight tendency for a decrease in the F_z_ values with 30 to 45 and 30 to 60 m/min increases in the cutting speed. The main reason for the reduction in F_z_ is that the cutting temperature increases with increasing cutting speed, facilitating material plastic deformation. Moreover, thinner chips are formed due to the increased slip angle with increasing cutting speed. This phenomenon is known to help reduce thrust by reducing the tool-to-chip contact length [14]. In particular, the decrease in the impact energy and tensile strength in samples with a high rate of GB reinforcement indicates poor bonding between the reinforcement and matrix material. However, an increased cutting speed results in tinier chip separations because of improved stress at the chip surface and the poor adhesive bond between the GF and polymer matrix [46]. This formation facilitated chip breaking, resulting in the formation of shorter chips and, consequently, a reduction of frictional pressure on the tool–chip surface. As a result, it is understood that the thrust forces reduce as the material strength decreases and as the chips are rapidly removed from the cutting medium. However, as can be seen from both graphs and the ANOVA results, the effect of the cutting speed on F_z_ is very low.

As can be noticed from Figure 4 and Table 3, the most influential parameter on the F_z_ is the feed rate. When the feed rate for each composite material (C1–C5) rises from 0.05 mm/rev to 0.1 mm/rev at the cutting speed of 30 m/min, the F_z_ values are approximately 24% and 32%, respectively, according to the material code. The changes in F_z_ values were obtained as 60%, 72%, 61%, 64%, and 71%, respectively, when increasing the feed rate from 0.05 to 0.15 mm/rev. At a cutting speed of 45 m/min, the increases in F_z_ values when the feed rate was increased from 0.05 to 0.1 mm/rev in each material were 23%, 35%, 28%, 31%, and 33%. The F_z_ values increased 65%, 77%, 56%, 68%, 71%, respectively, when the feed rate was increased by 200%. For a cutting speed of 60 m/min, increasing the feed rate by 100%, the F_z_ values were 27%, 42%, 32%, 33%, and 33%, respectively, and by increasing the feed rate by 200%, 67%, 84%, 73%, and 65%, it was determined that it increased by 74%. The highest change in F_z_ was obtained in GF-reinforced PA66 polymer material (C2) with 30%, and the lowest increase was obtained in the GB-reinforced PA66 polymer material (C1) with 30%. The complex fiber orientation distributions in the C2 composite due to the fiber reinforcement’s element shape and size, along with the use of the injection molding method, resulted in stronger interfacial shear strength [47]. 

It has ensured that this material has higher hardness, strength, and stiffness. As a result, the power required for machining the material increased, thereby increasing the thrust force. It is understood from the graphs that different reinforcement ratios and cutting speed changes have little impact on the F_z_ value, and the ANOVA results given in Table 3 confirm this situation. The ANOVA table and the percentage contribution of input parameters are shown in Table 3, showing that the material (M), cutting speed (V_c_), and feed rate (f) affected the thrust forces at 0.87%, 1.90%, and 96.94%, respectively. Based on these outcomes, the feed rate is the parameter that contributes the most to F_z_.

### 3.3. Assessment of Surface Texture by Surface Roughness (Ra)

**Figure 5:**
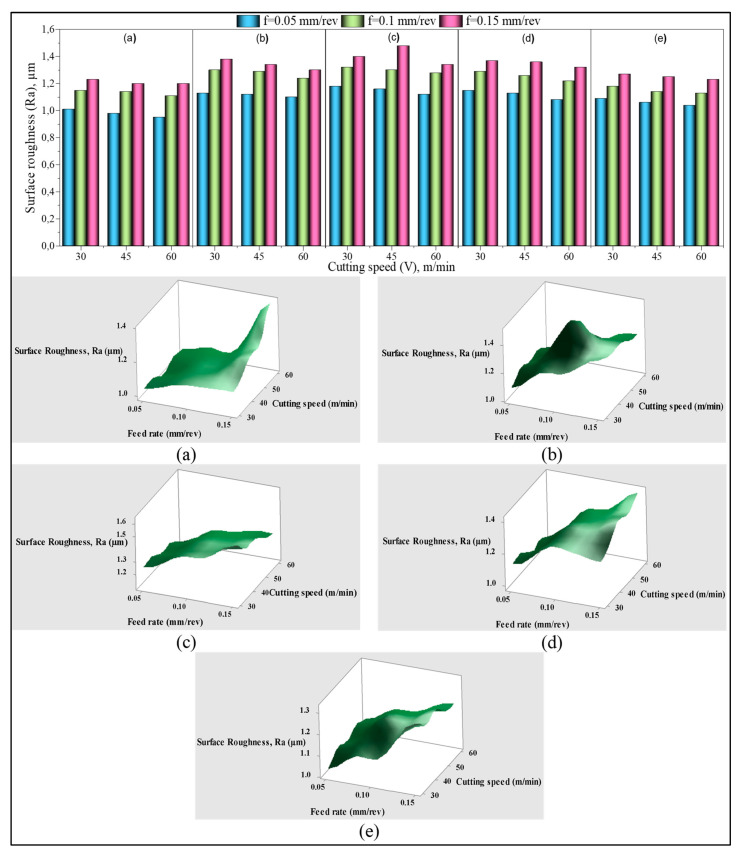
Surface roughness (Ra) variation versus drilling parameters with 2D bar and 3D surface plots: (**a**) C1, (**b**) C2, (**c**) C3, (**d**) C4, (**e**) C5.

The surface roughness of the holes machined in the GB, GF, and GB + GF mixed additive PA66 polymer materials is given in Figure 5. It can be seen that R_a_ decreased with the increase of cutting speed, and increased with the increase of the feed rate. Increasing the chip cross-section with an increase in feed means increased chip thickness, facilitating chip breaking [48]. Despite the ductile matrix, this process is thought to partially increase the surface roughness by promoting flaky chip formation. In addition, the presence of short fiber and glass bead reinforcement elements increases the strength of the material, causing it to exhibit a brittle behavior and support the formation of chopped chips. Therefore, these formations are the main reason for the increase in the surface roughness, and are similar to the results obtained in the literature [49,50]. It is seen that there is a decreasing tendency in R_a_ values with 50% and 100% increases in cutting speed. This result is expected, and it is known that the surface quality improves as the friction decreases due to the decrease in the tool–chip interface with rising cutting speed, or the cutting temperature decreases the material’s yield strength [51]. On the other hand, it is understood from the graphs that the cutting speed has no considerable influence on the R_a_ value, and the ANOVA results given in Table 4 confirm this situation. The ANOVA results presented in Table 4 show that “M”, “V_c_”, and “f” affected the R_a_ at 31.33%, 3.65%, and 63.59%, respectively. According to these results, while the feed rate was the main factor affecting R_a_, it was determined that the second important parameter was the material produced depending on the reinforcement ratio. 

**Figure 6:**
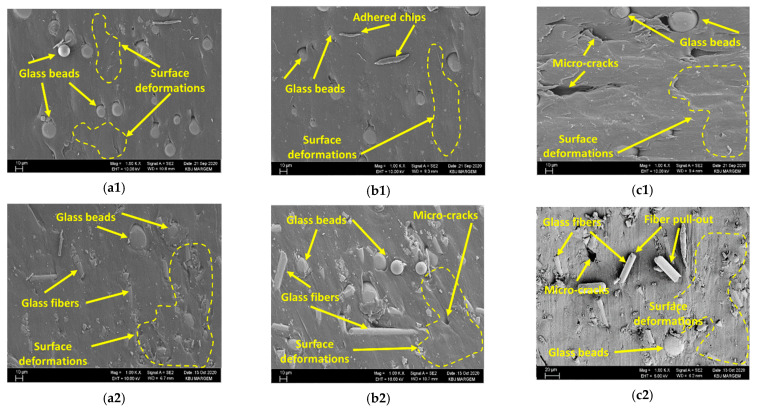
The hole surfaces at V = 45 m/min; (**a1**,**b1**,**c1**) C1 and (**a2**,**b2**,**c2**) C4 for from 0.05 mm/rev to 0.15 mm/rev, respectively.

When the R_a_ changes are assessed according to the feed rate (Figure 6), when the feed rate is increased from 0.05 to 0.1 mm/rev for each material (C1–C5) at 30 m/min cutting speed, the R_a_ values are calculated according to the material code. It was calculated that it increased by approximately 14%, 15%, 8%, 12%, and 13%, respectively. Increasing the feed rate in the same parameters from 0.05 to 0.1 mm/rev the changes in R_a_ values were obtained as 18%, 21%, 17%, 19%, and 22%, respectively. As mentioned in Ref. [52], these results reveal the dominant impact of the feed rate on R_a_, and the SEM pictures taken through the hole according to the increase of feed at a medium cutting speed between single reinforced (C1) and hybrid (C4) composites with the lowest R_a_ support these results (Figure 6). When the hole surfaces of both groups are examined, it is seen that the reinforcement elements come out of place or drift in the matrix with the increase of the feed rate, and as a result, a morphology that will deteriorate the surface quality is formed.

**Figure 7:**
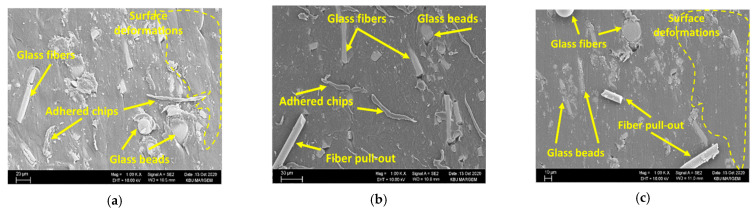
Hole surfaces of C3 for f = 0.05 mm/rev: (**a**) 30 m/min, (**b**) 45 m/min, (**c**) 60 m/min.

The R_a_ values showed a downward trend due to the increased cutting speed. However, these changes have a low impact relative to the feed rate, and range from 5% to −8%. Once the cutting speed is increased from 30 to 45 m/min at f = 0.05 mm/rev, the change varies between −1% and −3%. It was observed that this change ranged from 1% to 5% at the increase of cutting speed from 30 to 60 m/min. According to these findings, it can be said that the positive impact of rising cutting speed on R_a_ is seen more dominantly in low feed rate (0.05 mm/rev). This result is supported by the intra-hole SEM images of the C3 coded samples with the lowest Ra among the hybrid composites (Figure 7).

**Figure 8:**
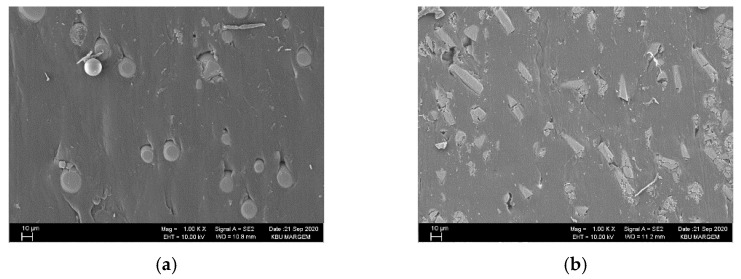

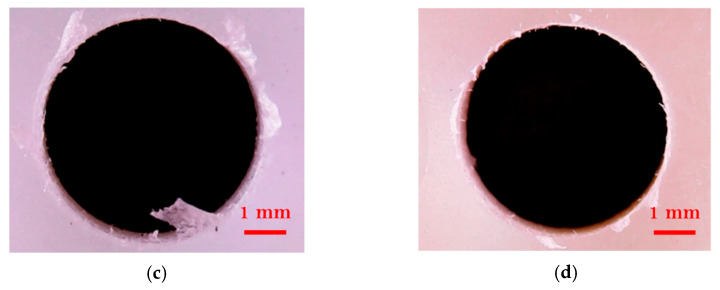
V = 45 m/min, f = 0.05 mm/rev: (**a**) C1 and (**b**) C2 hole surface SEM images, (**c**) C1 and (**d**) C2 hole exit images.

**Figure 9:**
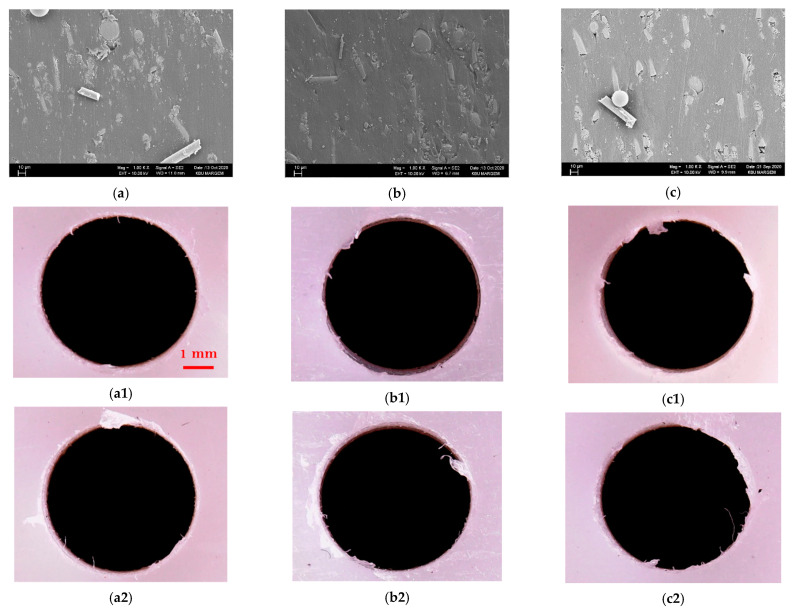
V = 45 m/min, f = 0.05 mm/rev: (**a**) C3, (**b**) C4, and (**c**) C5 SEM images for the hole surface, (**a1**,**b1**,**c1**) hole exit for f = 0.05 mm/rev, (**a2**,**b2**,**c2**) hole exit for f = 0.15 mm/rev.

Among the composites, the lowest surface roughness was obtained in the GB-reinforced materials (C1) under all cutting conditions. Compared to the GF-reinforced materials (C2), the Ra values for all cutting and feed rates were, on average, 12.3% lower. This result is thought to be due to the higher ductility and lower mechanical properties of the GB-reinforced material. That is, the reinforcement phase was easily dislodged during drilling, and afterward, the reinforcement phase formed in the hole walls of the matrix material was closed and the surface roughness was low (Figure 8a). Although the situation is similar in GF-reinforced composite, it is considered that the surface roughness is higher due to the complex distribution of the reinforcing elements [42]. On the other hand, in the GB-reinforced composite, it was determined that the more intense plowing–cutting process increased the delamination at the hole exit (Figure 8c). When the hybrid composites are evaluated, it is seen that the R_a_ values are similar to others in 20% GF + 10% GB and 15% GF + 15% GB materials, but the R_a_ values obtained from the 10% GF + 20% GB-reinforced mixed parts were lower than the others. From this, it is thought that the reduction of the matrix-reinforcement element bond strength as the GB reinforcement ratio increases facilitates the chip formation, while the cutting is in the form of scraping. In this case, as can be seen from the SEM pictures in Figure 9, the surface roughness was positively affected as a result of the plastering of the matrix material to cover the defects (voids, fiber pull out, etc.) on the hole surfaces. However, although the hole exit delaminations in the hybrid composites are close to each other, it appears that the amount of delamination develops with increasing feed rate (Figure 9). The main reason for this is because the rise in the feed rate increases the thrust force and temperature towards the hole exit, with a rise in the cutting speed causing severe matrix fragmentation [53,54].

The lowest R_a_ value among the PA66-based single reinforced composites was obtained with f = 0.05 mm/rev and V = 60 m/min in the sample reinforced with 30% GB. The highest R_a_ value was 0.38 µm in the sample with 30% GF additive at the feed rate of 0.15 mm/rev and the cutting speed of 30 m/min. Among the hybrid composites, the lowest R_a_ was 1.04 µm in 10% GF + 20% GB sample at 0.05 m/rev feed rate and 60 m/min cutting speed. The highest R_a_ was 1.4 µm at 30 m/min cutting speed and 0.15 m/rev feed rate in 20% GF + 10% GB material.

## 4. Conclusions

In this paper, the mechanical properties and machinability of single reinforced and hybrid glass bead/fiber-reinforced polymer composites (PA66) were analyzed. Firstly, polymer materials reinforced with different ratios were manufactured and their mechanical properties (tensile strength, hardness, modulus of elasticity) were evaluated. Then, fabricated samples were machined to assess the effect of the cutting parameters and reinforcement ratio on the thrust force and hole surface roughness. Scanning electron microscopy was used to characterize the fabricated materials and the quality of the machined holes. The following results can be concluded from this study:

- The sample reinforced with 30% GF has the highest strength, coefficient of elasticity, impact strength, and hardness. Impressive improvements were obtained for the strength—approximately 92% (142 MPa)—and the sample exhibited a coefficient of elasticity of approximately 186% (8390 MPa) compared to PA66.
- Among the six samples produced, the very best mechanical properties were obtained within the sample PA66 + 20% GF + 10% GB, with 127 MPa for lastingness and 7470 MPa for the modulus of elasticity.
- Thrust forces show an increasing trend of about 100–200% with higher feed rate values consistent with the graphs, while a decreasing tendency of about 50–100% is observed with increasing cutting speed. The ANOVA supports the experimental graphs that indicate that the feed rate is noticed because of the major factor on the thrust force (96.94%).
- Surface roughness indicates a decreasing tendency of about 50–100% with the rise in cutting speed. Additionally, a rise in the surface roughness is observed with the rise of 100–200% of the feed rate. This is consistent with the ANOVA results, which prove the validity of the graphs showing that material type (31.33%), cutting speed (3.65%), and feed rate (63.59%) affected the surface roughness at several levels.
- A major recommendation from this study is that a suitable combination should be preferred for the optimum machinability and mechanical properties of GF/GB-reinforced polymers instead of only using glass fibers or glass beads.

## Figures and Tables

**Table 1:** Drilling parameters and their levels.

Parameters	Levels				
**Cutting speed (m/min)**	30	45	60		
**Feed rate (mm/rev)**	0.05	0.1	0.15		
**% ratio of reinforcements**	30% GFR	30% GB	20% GFR + 10% GB	15% GFR + 15% GB	10% GFR + 20% GB

**Table 2:** Mechanical properties of GB/GF-reinforced PA66 matrix composites.

Materials	Tensile Strength, MPa	Young Modulus, MPa	Strain at Fracture %	Izod Impact Energy kJ/m^2^	Hardness (Shore D)
PA66 + 30% GB	58.9	4210	5.6	4.69	79.8
PA66 + 30% GFR	142	8390	5.1	7.46	82.9
PA66 + 20% GFR + 10% GB	127	7470	5.4	4.79	82.3
PA66 + 15% GFR + 15% GB	112.1	6203	5.3	5.1	81.7
PA66 + 10% GFR + 20% GB	96.9	5160	5.6	6.17	81

**Table 3:** ANOVA for thrust force.

Source	DoF	SS	MS	F	*p*	PCR (%)
**M**	4	97.30	24.33	47.10	0.000	0.87
**Vc**	2	213.30	106.67	206.45	0.000	1.90
**f**	2	10,904.10	5452.07	10,552.39	0.000	96.94
**M × Vc**	8	12.70	1.58	3.06	0.027	0.11
**M × f**	8	10.50	1.32	2.55	0.053	0.09
**V × f**	4	1.70	0.43	0.84	0.521	0.02
**Error**	16	8.30	0.52			0.07
**Total**	44	11,248.00				100.00

**Table 4:** ANOVA for surface roughness.

Source	DoF	SS	MS	F	*p*	PCR (%)
**M**	4	0.179347	0.044837	430.43	0.000	31.33
**Vc**	2	0.020938	0.010469	100.50	0.000	3.65
**f**	2	0.364084	0.182042	1747.61	0.000	63.59
**M × Vc**	8	0.000840	0.000105	1.01	0.467	0.15
**M × f**	8	0.005627	0.000703	6.75	0.001	0.98
**V × f**	4	0.000022	0.000006	0.05	0.994	0.01
**Error**	16	0.001667	0.000104			0.29
**Total**	44	0.572524				100

## Data Availability

Not applicable.
